# Assessment of Photo-Induced Cytotoxic Activity of *Cachrys sicula* and *Cachrys libanotis* Enriched-Coumarin Extracts against Human Melanoma Cells

**DOI:** 10.3390/plants10010123

**Published:** 2021-01-08

**Authors:** Mariangela Marrelli, Maria Rosaria Perri, Valentina Amodeo, Francesca Giordano, Giancarlo A. Statti, Maria Luisa Panno, Filomena Conforti

**Affiliations:** Department of Pharmacy, Health and Nutritional Sciences, University of Calabria, 87036 Rende, Cosenza, Italy; mariarosaria.perri@unical.it (M.R.P.); valentina.amodeo@unical.it (V.A.); francesca.giordano@unical.it (F.G.); mluisa.panno@unical.it (M.L.P.)

**Keywords:** Apiaceae, *Cachrys* spp., furanocoumarins, green extraction technology, photochemotherapy, skin cancer

## Abstract

Photochemotherapy is one of the most interesting current therapeutic approaches for the treatment of melanoma. Different classes of naturally occurring phytochemicals demonstrated interesting photoactive properties. The aim of this study was to evaluate the photocytotoxic potential of two *Cachrys* species from Southern Italy: *C. sicula* and *C. libanotis* (Apiaceae). The enriched-coumarin extracts were obtained from aerial parts through both traditional maceration and pressurized cyclic solid-liquid (PCSL) extraction using Naviglio extractor^®^. Qualitative and quantitative analyses of furanocoumarins were performed with GC-MS. The photocytotoxic effects were verified on C32 melanoma cells irradiated at a dose of 1.08 J/cm^2^. The apoptotic responses were also assessed. Moreover, phenolic content and the in vitro antioxidant potential were estimated. Xanthotoxin, bergapten, and isopimpinellin were identified. All the samples induced concentration-dependent photocytotoxic effects (IC_50_ ranging from 3.16 to 18.18 μg/mL). The *C. libanoti*s sample obtained with Naviglio extractor^®^ was the most effective one (IC_50_ = 3.16 ± 0.21 μg/mL), followed by *C. sicula* sample obtained with the same technique (IC_50_ = 8.83 ± 0.20 μg/mL). Both *Cachrys* samples obtained through PCSL induced up-regulation of apoptotic signals such as BAX (Bcl2-associated X protein) and PARP (poly ADP-ribose polymerase) cleavage. Moreover, these samples proved to be more photoactive, giving a greater upregulation of p21 protein in the presence of UVA radiation. Obtained results suggest that investigated species could be promising candidates for further investigations aimed to find new potential drugs for the photochemotherapy of skin cancer.

## 1. Introduction

The World Health Organization (WHO) indicates that cancer is a dominant public health issue with a high mortality rate. Among skin cancer, melanoma is the most aggressive form, with an average of 232,000 new cases/year [1]. It is possible to identify three types of skin cancer, whose names derive from the kind of involved cells: Basal cell carcinoma, squamous cell carcinoma, and malignant melanoma. Basal and squamous types belong to the group of non-melanocytic skin cancer while malignant melanoma is the most aggressive and metastatic type of skin cancer which develops from melanocytes [2].

MAP kinases and phosphatidylinositol-3-kinase (PI3K) are the major signal transduction pathways involved in the development of melanoma. Currently, there are many treatment strategies aiming at blocking the migratory and invasive capacity of cancer cells [3]. The cellular response to most drugs is usually associated with high cell resistance, mainly when the diagnosis is late. In this context, the search for natural products for the prevention and treatment of cancer has gained importance, since some phytochemicals have shown proapoptotic and antiangiogenic effects [4]. 

Natural products, depending on the molecular structure, present different mechanisms of action against tumor cells, such as inhibiting cell proliferation, promoting apoptosis, and inducing autophagy. Flavonoids, a group of biologically active polyphenolic compounds, have been found to play an important role in the inhibition of proliferation and in chemoprevention. Most bioactive flavonoids belong to the classes of flavones (38.0%), flavonols (17.5%), or isoflavonoids (17.5%) [5]. Different phytochemicals have been shown to possess photosensitizing activity and thereby to exert a greater anti-cancer action, since, during the photochemical reaction, the high production of local ROS directs cancer cells to die through apoptosis and/or necrosis [6].

Other authors have shown that coumarins are especially useful in blocking the growth of rapidly dividing tumoral cells [7].

The molecular studies about these products led to the identification of transductional signals, mainly involved in the regulation of cell survival of breast tumoral cells and targeted by these drugs [8,9].

Anticancer activity on human melanoma cells, due to cytotoxic effects, has been reported for components extracted from Polygonaceae and Asteraceae. Triterpenes and phenolic acids, their main metabolites, seem to be responsible for the cytotoxicity and apoptosis documented in the C32 cell line [10,11].

Similarly, antitumoral activity against human melanoma cells has been demonstrated for extracts from *C. pungens* Jan from Italy. Different coumarins were identified: The linear furanocoumarins xanthotoxin, bergapten, psoralen, isooxypeucedanin, and isopimpinellin, the angular furanocoumarin columbianetin, the dimethyl-pyranocoumarin jatamansin, and the coumarin 3-methylsuberosine. The methanolic extract, and particularly its chloroform fraction and isolated coumarins fraction, showed strong photocytotoxic activity on UVA irradiated A375 melanoma cells, with IC_50_ values equal to 0.487 ± 0.037, 0.286 ± 0.067, and 0.209 ± 0.033 µg/mL, respectively [12]. So, we focalized our attention on other *Cachrys* species in order to find new fractions with phototoxic activity against human melanoma cells: *C. sicula* L. and *C. libanotis* L. 

*Cachrys* genus (Apiaceae) consists of more than 100 species, and is widely distributed in the Mediterranean basin. Both *C. libanotis* and *C. sicula* are widely distributed in Southern Italy. *C. libanotis* is a perennial plant also present in the South West of Europe and North Africa. It’s between 40–80 cm in height; its leaves are divided into linear strips. Flowers are yellow and organized in umbel. Its fruits are smooth and ovoid (10–15 mm) and its seeds are extremely acrid. Its roots are gross, white, branching, and aromatic. *C. sicula* is of 3–15 dm height, the stem is erect, streaked, very branchy, for opposite or verticillated branches, reddened at the base. Its native range is western and central Mediterranean [13]. Very few studies have been carried out on these plants. Some coumarins and ferurol esters have been identified in *C. sicula* [14]. De Leo and coworkers isolated the three coumarins 8-hydroxymethylpsoralen, sprengelianin, and oxypeucedanin from the dried leaf extract [15]. Palá-Paúl et al. [16] also investigated the chemical composition of the essential oil of *C. sicula* aerial parts and reported the presence of many terpenes, while Pinar [17] isolated the diamine N-N′-di-o-tolylethylendiamine. Some photodermatitis agents have been identified in *C. libanotis* [18] and the presence of various terpenes have been detected in its essential oil [19]. 

In this study, we describe the phytochemical fingerprint, the antioxidant potential, and phototoxicity of enriched-coumarin extracts from these two *Cachrys* species, *C. sicula* and *C. libanotis*, obtained by two different extraction processes: Traditional maceration (TM) and pressurized cyclic solid–liquid (PCSL) extraction. This last process has been carried out using the Naviglio extractor^®^, a rapid and innovative solid–liquid dynamic extraction method, which allows us to reduce the extraction times compared to classical maceration [20,21]. The mechanism of action of phototoxicity is also described.

## 2. Results

### 2.1. Phytochemical Profile

In this work, aerial parts of *C. sicula* and *C. libanotis* species from Southern Italy were extracted with methanol through two different extraction techniques: Traditional maceration (TM) and pressurized cyclic solid–liquid (PCSL) extraction. As regards the raw extracts obtained through PCSL technique, achieved yields were 7.05% and 12.56% for *C. sicula* L. (CSN) and *C. libanotis* L. (CLN), respectively (Table 1). Higher yields were instead obtained with the classical maceration method: 19.48% and 17.76 for *C. sicula* (CSM) and *C. libanotis* (CLM), respectively. 

Total phenolic and total flavonoid contents of *Cachrys* spp. raw extracts were expressed as equivalents of chlorogenic acid or quercetin per g of dry plant material, respectively. The highest amounts were detected in the extracts obtained through maceration. CLM showed the highest amounts of phenolic compounds (25.0 ± 0.2 mg/g, Table 1) and flavonoids (1.29 ± 0.04 mg/g), followed by CSM (23.1 ± 0.7 and 0.65 ± 0.04 mg/g). Lower amounts were observed for the other two samples both for phenolic content (12.8 ± 0.1 and 11.4 ± 0.2 mg/g for CLN and CSN, respectively) and flavonoid content (0.09 ± 0.01 and 0.41 ± 0.02 mg/g). 

**Figure 1:**
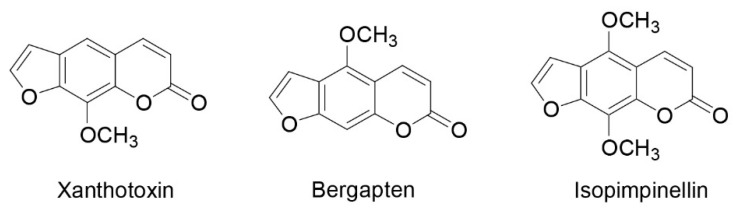
Structure of identified furanocoumarins.

Samples were then chemically characterized by GC-MS (gas-chromatography coupled to mass spectrometry). Compounds were identified according to their mass spectra and retention times. Eight coumarins were identified (Table 2), including three furanocoumarins: Xanthotoxin, bergapten, and isopimpinellin (Figure 1).

The pyranocoumarin compound 2-methyl-2-butenoic acid 9,10-dihydro-8,8-dimethyl-2-oxo-2H,8H-benzo[1,2-b:3,4-b*′*]dipyran-9-yl ester was detected only in the Naviglio extracts (about 10% of total peak areas), while seselin was identified just in CLM, which also contained three coumarins: osthol, suberosin, and isogeijerin. Moreover, three different fatty acids were recognized, palmitic acid being the major one.

*C. sicula* also demonstrated a higher content in terpenes: Limonene, fenchone, estragole, anethole, and neophytadiene were identified. On the contrary, estragole was the only compound found in *C. libanotis*.

Given the importance of furanocoumarins for the phototoxic potential of investigated samples, the quantification of the three psoralens xanthotoxin, bergapten, and isopimpinellin was then accomplished (Table 3).

Xanthotoxin was the most abundant compound (concentration ranging from 2.08 to 4.98 mg/mL). CLN showed the highest content (4.98 ± 0.21 mg/mL), followed by CSN obtained through the same extraction method (4.10 ± 0.23 mg/mL), while traditional maceration allowed to reach lower amounts. A similar trend was observed for bergapten, with a higher content in CLN and CSN (0.59 ± 0.08 and 0.58 ± 0.04 mg/mL, respectively) compared to CLM and CSM (0.27 ± 0.02 and 0.17 ± 0.01 mg/mL, *p* < 0.05, Bonferroni post-hoc test). No statistical differences were detected among samples as regards the isopimpinellin content.

### 2.2. Photocytotoxic Activity

**Figure 2:**
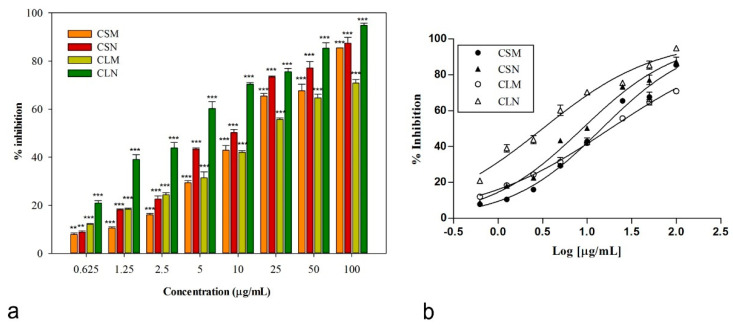
(**a**) Concentration-dependent photocytotoxic effects induced by *Cachrys* samples. Cells were irradiated for 1 h at a dose of 1.08 J/cm^2^; (**b**) non-linear regression analyses: CSM, *C. sicula* L. maceration; CSN, *C. sicula* L. Naviglio extractor^®^; CLM, *C. libanotis* L. maceration, CLN, *C. libanotis* L. Naviglio extractor^®^. Data were expressed as means ± SEM (*n* = 4). *** *p* < 0.001, ** *p* < 0.01 (Dunnett’s test) compared to control (untreated irradiated cells).

To evaluate the anticancer activity of *Cachrys* samples on melanoma, C32 cells (human melanoma) were treated with different concentrations of each sample for 30 min of incubation and 1 h of irradiation, and cell viability was determined after 48 h. All samples from the two *Cachrys* species, obtained with two extraction methods, affected cell viability in a concentration-dependent manner after irradiation for 1 h at a dose of 1.08 J/cm^2^ (IC_50_ ranging from 3.16 to 18.18 μg/mL, Figure 2, Table 4).

Obtained results showed that the extraction with Naviglio extractor^®^ allowed a better chemical composition for the antitumor activity than the traditional maceration technique. The best activity was observed for CLN sample (*C. libanotis* Naviglio), with an IC_50_ of 3.16 μg/mL, a very interesting value if compared with the positive control bergapten (*p* < 0.05, Bonferroni post-hoc test). The *C. sicula* sample obtained with Naviglio extractor^®^ also showed a very good inhibitory activity on cell line viability (IC_50_ = 8.83 ± 0.20 μg/mL). Even if both these samples, at the highest concentration tested, also induced cytotoxic effects in the dark, the IC_50_ values observed for unirradiated cells were significantly higher than those referred to UVA-treated plates (IC_50_ values equal to 55.20 ± 1.65 and 26.72 ± 1.18 μg/mL for CLN and CSN, respectively). A lower, but still noteworthy, photocytotoxicity was exerted by the two extracts obtained through maceration (IC_50_ values of 14.57 ± 0.95 and 18.18 ± 1.33 μg/mL, for *C. sicula* and *C. libanotis*, respectively), which did not affect cell viability in the dark.

**Figure 3:**
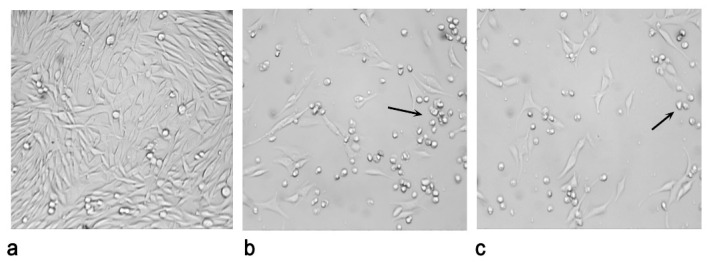
C32 cells morphology 48 h after UVA irradiation at a dose of 1.08 J/cm^2^. (**a**) Control, irradiated cells in RPMI 0.5% MeOH, without sample; (**b**) irradiated cells, *C. sicula* L. obtained with Naviglio extractor^®^, 25 μg/mL; (**c**) irradiated cells, *C. libanotis* L. extracted through Naviglio extractor^®^, 25 μg/mL. Cells were visualized with an inverted microscope AE20 Motic and images were captured with a VisiCam digital camera. Arrows show cells became rounded and shrunken. Magnification, 10×.

Figure 3 shows cell morphology 48 h after UVA irradiation as captured on a digital camera. The incubation of cell cultures with a concentration of 25 µg/mL of both *C. sicula* and *C. libanotis* PCSL samples and UVA radiation significantly affected cell viability compared to control (untreated irradiated cells).

### 2.3. Apoptotic Responses on C32 Cells

**Figure 4:**
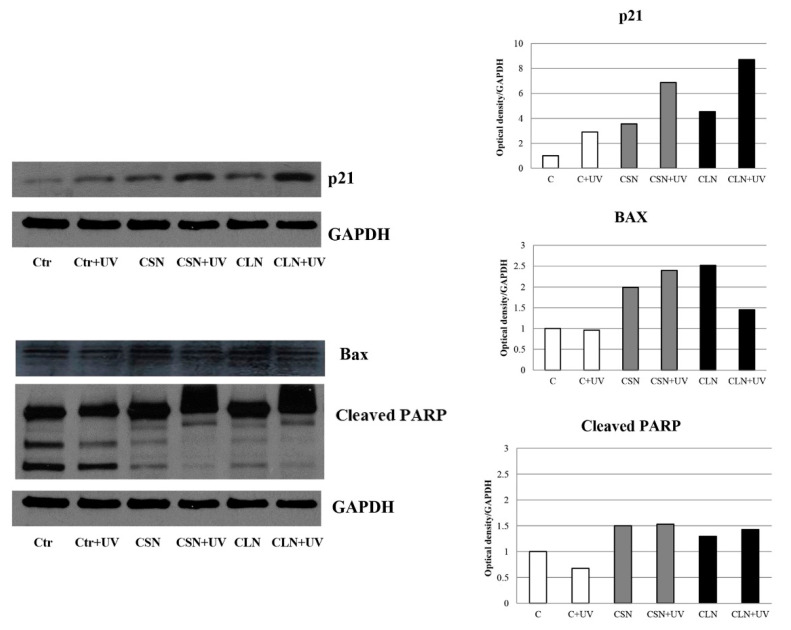
Western Blot analysis of p21, BAX and PARP (poly ADP-ribose polymerase) protein levels in C32 cells treated or not with CSN and CLN extracts for 24 h, both in the presence and absence of UV. The histograms refer to the densitometric analysis (OD) of the Western blot shown in the figure.

The two *Cachrys* species were also evaluated to assess apoptotic responses on C32 cells. The Naviglio extracts CSN and CLN increase the cyclin-dependent kinase inhibitor p21 protein, with respect to control, with greater up-regulation under the combination with UV. Both CSN and CLN samples stimulate the pro-apoptotic BAX protein. Consistently, PARP (poly ADP-ribose polymerase) signature fragments, biomarkers of cell death, are specifically induced under CSN and CLN, with or without UV exposure (Figure 4).

**Figure 5:**
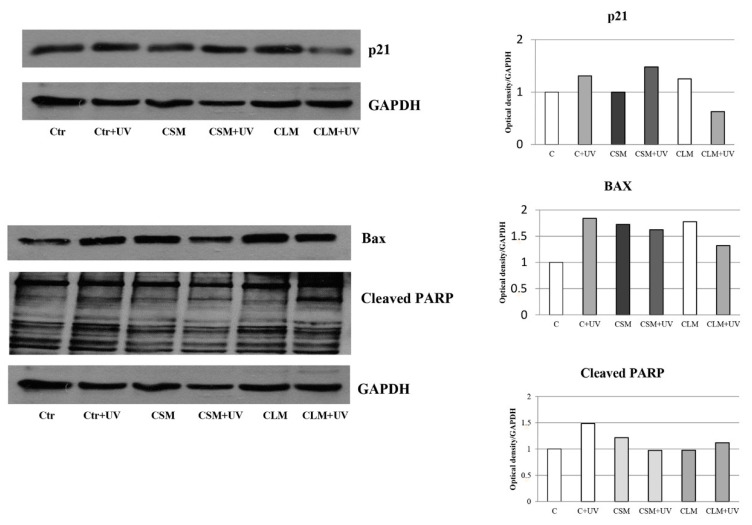
Western Blot analysis of p21, BAX, and PARP (poly ADP-ribose polymerase) protein levels in C32 cells treated or not with CSM and CLM extracts for 24 h, both in the presence and absence of UV. The histograms refer to the densitometric analysis (OD) of the Western blot shown in the figure.

The other *Cachrys* samples, CSM and CLM, do not elicit the same results mentioned above. In fact, no increase of p21 protein levels are evidenced in C32 cells that underwent treatment with the maceration products of the *Cachrys*, and if anything, under UV there is a reduction of the protein with respect to single treatment and control. In contrast, the same treatments cause a significant up-regulation of BAX, the levels of which remain equally high in the presence of co-treatment with UV rays, no significant PARP cleavage is evident under the same experimental conditions (Figure 5).

### 2.4. In Vitro Antioxidant Activity

Furthermore, the in vitro antioxidant activity of the four *Cachrys* extracts was evaluated. Two methods were employed: The DPPH and the β-carotene bleaching test. All the *Cachrys* samples demonstrated concentration-dependent radical scavenging potency. The two traditional alcoholic macerates (IC_50_ = 102.13 ± 0.79 and 112.73 ± 0.88 μg/mL for CLM and CSM, respectively, Table 5) exerted a better radical scavenging potency than two samples obtained through PCSL extraction (IC_50_ = 163.80 ± 2.63 and 212.80 ± 6.91 μg/mL, *p* < 0.05, Bonferroni post-hoc test).

The same samples, CLM and CSM, also demonstrated an interesting antioxidant activity in the β-carotene bleaching method, with IC_50_ values equal to 16.77 ± 1.43 (CSM) and 19.22 ± 1.07 μg/mL (CLM) after 30 min of incubation. After 60 min, these extracts were still effective, with IC_50_ values of 31.07 ± 1.71 and 27.52 ± 1.73 μg/mL. The samples obtained though Naviglio extractor^®^ were also effective, even if to a lesser extent.

## 3. Discussion

Among skin cancer with rapid growth, the melanoma represents the most invasive and metastatic one. Due to its strong aggressiveness, there are no effective treatments to antagonize cell growth, and often the cancer cells over time develop resistance to chemotherapy. In patients with metastatic melanoma, several immune therapies have been approved, however, with unsatisfactory responses compared to those obtained with targeted therapies [22].

In this context, more recently, there is the great attention towards natural products since they have been useful in antagonizing chemotherapy resistance as well as they lack major side effects [23].

In the present paper, we investigated the effect of two species of *Cachrys* (*C. sicula* and *C. libanotis*), subjected to different extraction processes, on the apoptotic response of the C32 human melanoma cell line.

In particular, as previously reported, by comparing two methods of extraction on aerial parts of *Cachrys*, the results indicated that pressurized cyclic solid–liquid (PCSL) extraction yields biologically more active products since they address significant apoptotic effects in C32 cells than the traditional maceration (TM).

The solid–liquid dynamic extractor is an innovative technology of solid–liquid extraction that allows to carry out the extraction in a short time, compared to the other currently used techniques. While most of them are based on osmosis and diffusion principles, PCSL works on Naviglio’s Principle, based on an increase of pressure of the extracting liquid on the solid plant material and the generation of a negative gradient pressure from the inside to the outside of the plant matrix [20,21].

In line with the other results discussed above, the CSN and CLN extracts are able to increase p21 protein, a well-known cyclin-dependent kinase inhibitor (CKI), that is able to affect all cyclin/CDK complexes [24]. Therefore, *Cachrys* that underwent the PCSL extraction technique is really stronger against C32 cells. In fact, *C. libanotis* and *C. sicula* extracted with Naviglio extractor^®^ lead proliferative arrest and up-regulation of apoptotic signals such as BAX and PARP cleavage. In addition, the above extracts are more photosensitive since in the presence of UV rays they give a greater upregulation of p21, while the increase in BAX and PARP remain equally sustained, like in the absence of photoactivation.

Thus, malignant melanoma C32 cell line was less responsive to apoptotic induction when incubated with *Cachrys* samples underwent to traditional maceration (TM), than extracts by Naviglio extractor^®^.

These data confirm those of cellular phototoxicity, in which it has been seen that the Naviglio extractor^®^ allows us to obtain a better chemical composition of *Cachrys* for its antitumor activity. Similarly, gas chromatography–mass spectrometry analyses allowed us to identify eight coumarins, including three furanocoumarins, the class of compounds known for their ability to act as photosensitizers [25]. All the four *Cachrys* samples were particularly rich in these tricyclic aromatic compounds made of a furan ring fused to a coumarin (α-benzopyrone) system: Xanthotoxin, bergapten, and isopimpinellin were detected, and quantitative analyses were also carried out. The exact molecular mechanism of these compounds relies upon their chemical structure, which in turn depends on the combination of the furan ring and coumarin backbone in an angular or linear structure, and on the number, type, and location of the attached substituents [26]. The CH_3_ presence at C5 and C8 improves the tumor properties [27]. The *C. libanotis* extract obtained through PCSL technique using Naviglio extractor^®^ (CLN) showed the highest content of xanthotoxin, (CH_3_ at C8) followed by *C. sicula* obtained through the same extraction method.

Samples CLN and CSN also showed a higher content in bergapten (CH_3_ at C5). No statistical differences were observed among the samples for the isopimpinellin content.

On the contrary, the two *Cachrys* macerates showed higher amounts of polar constituents. For both *Cachrys* species, these extracts showed a higher phenolic content compared to the extracts obtained through PCSL technique, with amounts equal to 25.0 ± 0.2 mg/g and 23.1 ± 0.7 for CLM and CSM, respectively. The same trend was observed for the total flavonoid content.

Consistently, samples obtained through maceration showed a better antioxidant activity, evaluated by the DPPH radical scavenging assay and the β-carotene bleaching test. Even if all the four *Cachrys* extracts demonstrated concentration-dependent biological activity, the two alcoholic macerates (IC_50_ values equal to 102.13 ± 0.79 and 112.73 ± 0.88 μg/mL) exerted a better radical scavenging potency than the other two samples (IC_50_ = 163.80 ± 2.63 and 212.80 ± 6.91 μg/mL), and no significant statistical differences were observed among the two species for this kind of activity. The same trend was maintained in the β-carotene bleaching test, with macerates being significantly more effective than samples realized with PCSL extraction.

In conclusion, the obtained results showed that extraction with the Naviglio extractor^®^ allowed a better chemical composition for the antitumor activity than the traditional maceration technique, and suggest that the investigated species could be promising candidates for further studies with the aim to find new potential drugs useful in the photochemotherapy of skin cancer. Furanocoumarins, probably, are the compounds directly involved in this activity, but the most important aspect is how these compounds are present together in the tested sample showing a high activity.

## 4. Materials and Methods

### 4.1. Reagents

Chlorogenic acid, ascorbic acid, Folin-Ciocalteu reagent, 2,2-diphenyl-1-picrylhydrazyl (DPPH), β-carotene, linoleic acid, Tween 20, fetal bovine serum (FBS), L-glutamine, penicillin/streptomycin, RPMI-1640 medium, trypan blue, phosphate buffered saline (PBS), Hanks’ Balanced Salt Solution, 3-(4,5-dimethylthiazol-2-yl)-2,5-diphenyltetrazolium bromide (MTT), and reference compounds were obtained from Sigma-Aldrich S.p.a. (Milano, Italy). Melanoma C32 cells were obtained from Type Culture Collection (ATCC) no. CRL-1585. p21, Bax, PARP, GAPDH, and peroxidase-coupled goat anti-mouse or goat anti-rabbit antibodies were obtained from Santa Cruz Biotechnology (Heidelberg, Germany); ECL System (Amersham Pharmacia Biotech, Cologno Monzese, Italy). All other reagents, were supplied by VWR International s.r.l. (Milan, Italy).

### 4.2. Plant Materials and Extraction Procedures

The aerial parts of *C. sicula* and *C. libanotis* were collected in Southern Italy. Plants were identified by Filomena Conforti, and a voucher specimen has been deposited at the Herbarium of natural History Museum of Calabria. The air-dried aerial parts of *C. sicula* and *C. libanotis* were extracted with methanol through maceration (72 h × 3 times, plant to solvent ratio 1:10 g/mL) and also by means of Naviglio extractor^®^ (Atlas Filtri SrL, Limena, PD, Italy) (plant:solvent ratio 1:10 g/mL × 2 cycles). Obtained total extracts (CSM and CLM for samples obtained with maceration; CSN and CLN for samples obtained with Naviglio) were concentrated under reduced pressure at 40 °C. The extracts were dried, weighed, and stored at 4 °C for experimental use.

### 4.3. Determination of Total Phenolic and Flavonoid Content

The total phenolic content was determined using the Folin-Ciocalteu method [28]. Briefly, 50 mg of the extracts were dissolved in 25.0 mL of an extraction solution (acetone: methanol: water: acetic acid, 40:40:20:0.1) and heated for 1 h at 60 °C. Then 200 μL of samples, 1.0 mL of Folin-Ciocalteu’s reagent and 1.0 mL of sodium carbonate (7.5%) were mixed and absorbance was measured at 726 nm 2 h later. The total phenolics content was calculated from the calibration curve, and the results were expressed as mg of chlorogenic acid equivalents per g of dried plant material.

The total flavonoid content of extracts was determined by a colorimetric method based on the use of aluminum chloride [29]. One mL of each extract (2 mg/mL in 80% EtOH) was added to 1 mL of 2% AlCl_3_. The absorbance was measured at 430 nm 15 min later. Results were calculated from calibration curve of the standard quercetin, and data were reported as mg of standard equivalent per g of dry plant material.

### 4.4. GC-MS Analysis

The coumarins, terpenes, and fatty acids content was assessed by means of gas chromatography–mass spectrometry (GC–MS). Qualitative GC-MS analyses were carried out using a gas chromatograph (Hewlett-Packard 6890) equipped with an SE-30 capillary column coupled to a selective mass detector (Hewlett-Packard 5973). Helium was utilized as the carrier gas, and a programmed temperature from 60 to 280 °C (with a rate of 16°/min) was used to realize the analyses. The injector and detector were set, respectively, at 250° and 280 °C [30]. Compounds were identified by comparing the GC retention factors with those of standards and the mass spectra with the Wiley 138 library data.

Quantitative GC analyses were performed to determine the content of furanocoumarins xanthotoxin, bergapten, and isopimpinellin in the extracts. Bergapten (10-0.16 mg/mL in MeOH/CHCl_3_) was used as the external standard, and the linear regression equation was

y = 1E + 07x + 2E + 06 (R^2^ = 0.9949), (1)

1 µL of each tested extract was injected in the GC-MS system at a final concentration of 50 mg/mL. All the analyses were carried out in isothermal conditions at 235 °C, and GC peaks area was determined in triplicate.

### 4.5. Cellular Phototoxicity

The photocytotoxic activity was determined as earlier described [31]. Human melanoma cells (C32) were grown in RPMI-1640 medium supplemented with L-glutamine, penicillin/streptomycin, and fetal bovine serum (1%, 1%, and 10%, respectively). For the experiments, 100 μL of medium containing 3.8 × 10^4^ cells were placed in each well of a 96-well tissue culture microtiter plate. After 24 h incubation, the medium was removed and 100 μL of samples (0.625–100 μg/mL), dissolved in MeOH and diluted with Hanks’ Balanced Salt Solution (HBSS, pH 7.2), were added to each well. Plates were incubated at 37 °C for 30 min and then irradiated. UV irradiation was carried out at 365 nm with an HPW 125 Philips lamp. The spectral irradiance of the source was 0.3 mW cm^−2^ as measured by a radiometer equipped with a 365-CX sensor (Cole-Parmer Instrument Company, Niles, IL, USA). Cells were irradiated for 1 h at a dose of 1.08 J/cm^2^. To prevent contamination from other light sources, the assay was performed in a dark room. Then, the solution was replaced with new medium, and microtiter plates were incubated for further 48 h. Bergapten was used as positive control.

Cytotoxicity was evaluated using the 3-[4,5-dimethyl-2-yl]-2,5-diphenyl tetrazolium bromide (MTT) assay [32]. Cell morphology was visualized after and without irradiation with an inverted microscope (AE20 Motic, Motic Instruments, Inc., VWR, Milano, Italy), and images were captured with a digital camera (VisiCam 3.0 USB, VWR, Milano, Italy).

### 4.6. Immunoblotting Analysis

The melanoma cells (C32) were harvested and lysed in 500 µL of RIPA buffer for total protein extraction. A 10% SDS-polyacrylamide gel was used for the resolution of the proteins that were transferred to a nitrocellulose membrane. Then, the filter was probed with p21, BAX, PARP, and GAPDH antibodies (Santa Cruz, Biotechnology, Heidelberg, Germany). The antibody antigen complex was detected with a secondary antibody conjugated to horseradish peroxidase and revealed with the ECL System (Amersham Pharmacia Biotech, Cologno Monzese, Italy) [33].

### 4.7. Free Radical Scavenging Activity (FRSA) Assay

The hydrogen atom or electron-donation ability of the extracts was evaluated based on the bleaching of a purple solution of 1,1-diphenyl-2-picryl-hydrazyl (DPPH), as earlier described [34]. Briefly, 200 μL of each extract (5–1000 μg/mL) were added to 800 µL of a 0.1 mM solution of DPPH and incubated in the dark. The absorbance was measured at 517 nm 30 min later. Ascorbic acid was used as a positive control.

### 4.8. β-Carotene-Linoleate Bleaching Assay

Antioxidant activity was determined using the β-carotene bleaching method [35]. β-carotene (0.2 mg in 1 mL chloroform) was added to 0.02 mL of linoleic acid and 0.2 mL of 100% Tween 20. Chloroform was removed and 100 mL of water were added. Then, 5 mL of the emulsion were added to 0.2 mL of samples in methanol (concentrations ranging from 0.25 to 100 μg/mL) and placed in a water bath at 45 °C for 60 min. Propyl gallate was used as standard. The absorbance was measured at 470 nm at the initial time and after 30 and 60 min. The antioxidant activity was measured in terms of the successful prevention of β-carotene bleaching.

### 4.9. Statistical Analysis

Experiments involving cell lines were carried out in quadruplicate, while the other assays were run in triplicate. Results were expressed as means ± SEM. Data were checked for normality and homogeneity of variances (D’Agostino-Pearson’s K2 test and Levene’s test, respectively). Biological data were fitted through nonlinear regression in order to calculate the IC_50_ values using GraphPad Prism Software (San Diego, CA, USA).

Statistical differences between treated groups and the control were estimated by one-way analysis of variance (ANOVA) followed by Dunnett’s multiple comparison test, while the statistical significance of differences among treated group means were tested by Bonferroni post-hoc test (*p* ≤ 0.05).

## Figures and Tables

**Table 1:** Investigated *Cachrys* species: Yields and total phenolics and flavonoids.

Sample	Extraction Technique	Abbreviation	Yield (%)	TP ^1^	TF ^2^
*C. sicula* L.	Maceration	CSM	19.48	23.1 ± 0.7 ^b^	0.65 ± 0.04 ^b^
	Naviglio^®^	CSN	7.05	11.4 ± 0.2 ^d^	0.41 ± 0.02 ^c^
*C. libanotis* L.	Maceration	CLM	17.76	25.0 ± 0.2 ^a^	1.29 ± 0.04 ^a^
	Naviglio^®^	CLN	12.56	12.8 ± 0.1 ^c^	0.09 ± 0.01 ^d^

^1^ Total phenolic content. ^2^ Total flavonoid content. Data are expressed as mean ± SD (*n* = 3). Results are expressed as mg of chlorogenic acid (for phenolics) or quercetin equivalent (for flavonoids) per g of dry plant material. Different letters along columns indicate statistically significant differences at *p* < 0.05 (Bonferroni post-hoc test).

**Table 2:** Phytochemical composition of *Cachrys* spp. extracts.

Compound	RT ^1^	CSM	CSN	CLM	CLN
		RAP ^2^			
**Furanocoumarins**					
Xanthotoxin	19.154	5.3	10.8	9.1	14.8
Bergapten	19.354	1.8	2.6	2.8	2.5
Isopimpinellin	20.571	1.6	3.1	3.4	3.0
**Pyranocoumarins**					
Seselin	19.462	-	-	0.6	-
2-Methyl-2-butenoic acid 9,10-dihydro-8,8-dimethyl-2-oxo-2H,8H-benzo[1,2-b:3,4-b′]dipyran-9-yl ester	24.423	-	10.2	-	9.7
**Coumarins**					
Osthol	19.822	-	-	2.8	-
Suberosin	20.388	-	-	2.7	-
Isogeijerin	21.154	-	-	1.2	5.6
**Fatty acids**					
Myristic acid	16.496	-	-	-	0.2
Palmitic acid	18.085	1.4	0.8	2.2	1.8
α-Linolenic acid	19.897	-	-	0.7	-
**Terpenes**					
Limonene	8.506	Tr ^3^	0.2	-	-
Fenchone	9.603	-	0.2	-	-
Estragole	11.141	0.2	0.8	0.1	0.7
Anethole	12.284	-	0.2	-	-
Neophytadiene	17.405	0.2	-	-	-

^1^ Retention time (as min). ^2^ Relative area percentage (in TIC %). ^3^ Traces < 0.1%.

**Table 3:** Quantitative analysis of identified furanocoumarins.

Species	Extract	Xanthotoxin	Bergapten	Isopimpinellin
	mg/mL ± SD			
*Cachrys sicula* L.	CSM	2.08 ± 0.04 ^c^	0.17 ± 0.01 ^b^	0.27 ± 0.01 ^a^
	CSN	4.10 ± 0.23 ^b^	0.58 ± 0.04 ^a^	0.75 ± 0.03 ^a^
*Cachrys libanotis* L.	CLM	2.23 ± 0.14 ^c^	0.27 ± 0.02 ^b^	0.46 ± 0.04 ^a^
	CLN	4.98 ± 0.21 ^a^	0.59 ± 0.08 ^a^	0.42 ± 0.03 ^a^

Data were expressed as mean ± SD (*n* = 3). Different letters along each column indicate statistically significant differences at *p* < 0.05 (Bonferroni post-hoc test).

**Table 4:** Photocytotoxicity of *Cachrys libanotis* L. and *Cachrys sicula* L. extracts on C32 cells.

Species	Extract	IC_50_ (μg/mL)	
		Irradiated Cells	Unirradiated Cells
*Cachrys sicula* L.	CSM	14.57 ± 0.95 ^c^	>100
	CSN	8.83 ± 0.20 ^b^	26.72 ± 1.18 ^d^
*Cachrys libanotis* L.	CLM	18.18 ± 1.33 ^c^	>100
	CLN	3.16 ± 0.21 ^a^	55.20 ± 1.65 ^e^
Bergapten ^1^		0.191 ± 0.012 ^a^	n.d.

Data were expressed as mean ± SEM (*n* = 4). Melanoma cells were pre-treated (30 min) with samples and then irradiated for 1 h at a dose of 1.08 J/cm^2^. Different letters indicate statistically significant differences at *p* < 0.05 (Bonferroni post-hoc test). ^1^ Positive control. n.d.: not detectable.

**Table 5:** Antioxidant activity of *Cachrys libanotis* L. and *Cachrys sicula* L. extracts.

Species	Extract	IC_50_ (μg/mL)		
		DPPH Test	β-Carotene Bleaching Test	
			30 min	60 min
*Cachrys sicula* L.	CSM	112.73 ± 0.88 ^b^	16.77 ± 1.43 ^b^	31.07 ± 1.71 ^c^
	CSN	163.80 ± 2.63 ^c^	48.62 ± 4.05 ^d^	68.55 ± 2.33 ^e^
*Cachrys libanotis* L.	CLM	102.13 ± 0.79 ^b^	19.22 ± 1.07 ^b,c^	27.52 ± 1.73 ^c^
	CLN	212.80 ± 6.91 ^d^	81.20 ± 1.52 ^f^	92.44 ± 1.08 ^g^
Ascorbic acid ^1^		2.00 ± 0.01 ^a^	-	-
Propyl gallate ^1^		-	1.00 ± 0.02 ^a^	1.00 ± 0.02 ^a^

Data were expressed as mean ± S. E. M. (*n* = 3) Statistically significant differences at *p* < 0.05 (Bonferroni post-hoc test) are indicated by different letters along (DPPH) or between columns (β-carotene bleaching test). ^1^ Positive controls.

## Data Availability

The data presented in this study are available on request from the corresponding authors.

## Abbreviations

CSM	*Cachrys sicula* extracted through maceration
CSN	*Cachrys sicula* extracted with Naviglio extractor^®^
CLM	*Cachrys libanotis* extracted through maceration
CLN	*Cachrys libanotis* extracted with Naviglio extractor^®^
PCSL	Pressurized cyclic solid-liquid extraction
TM	Traditional maceration
